# First analysis of *Spirometra mansoni* excretory–secretory proteins by the 4D-DIA method

**DOI:** 10.1051/parasite/2026025

**Published:** 2026-04-22

**Authors:** Fei Gao, Xuan Xuan Song, Jie Hao, Cheng Yue Cao, Shuang Li, Xi Zhang

**Affiliations:** Department of Parasitology, School of Basic Medical Sciences, Zhengzhou University Zhengzhou 450052 PR China

**Keywords:** *Spirometra mansoni*, Plerocercoid, Excretory–secretory protein, Comparative proteomics, Immune response

## Abstract

In this study, adults and plerocercoids of *S. mansoni* were cultivated *in vitro* to systematically analyze the components of the excretory–secretory proteins (ESPs) of *Spirometra mansoni*. Afterwards, the differentially expressed proteins (DEPs) were identified and protein components were examined using the Data Independent Acquisition (DIA) mode. A total of 944 proteins were identified, including 580 plerocercoid-specific proteins, whereas no specific proteins were found in adults. Quantitative analysis revealed that 607 proteins were significantly differentially expressed, with 390 upregulated in the plerocercoid group, and 217 upregulated in the adult group. Gene Ontology functional annotation revealed that the upregulated proteins in the plerocercoid group were significantly enriched in functions such as nitrogen compound metabolism, proteasome core complexes, and ion binding. Kyoto Encyclopedia of Genes and Genomes pathway enrichment revealed that the DEPs were strongly correlated with signal transduction, signal transportation, and catabolism pathways. Moreover, metabolic network analysis revealed that key pathways included the pentose phosphate pathway and glycolysis/gluconeogenesis. In addition, indirect ELISA revealed that immunization of mice with ESPs induced a Th1/Th2 mixed immune response, dominated by a Th1 response. Cytokine detection further verified that ESPs had good immunogenicity, and could activate both humoral and cellular immune responses. This study revealed, for the first time, the differential expression profile of ESPs between adults and plerocercoids of *S. mansoni*. These findings offer a potential reference for the diagnosis and prevention of sparganosis.

## Abstract (fr)

Afin d'analyser systématiquement les composants des protéines d'excrétion-sécrétion (PES) de *Spirometra mansoni*, les adultes et les plérocercoïdes de *S. mansoni* ont été cultivés *in vitro*. Les protéines différentiellement exprimées (PDE) ont ensuite été identifiées et leurs composants analysés par la méthode d'acquisition de données indépendante (DIA). Au total, 944 protéines ont été identifiées, dont 580 spécifiques aux plérocercoïdes, tandis qu'aucune protéine spécifique n'a été trouvée chez les adultes. L'analyse quantitative a révélé que 607 protéines étaient significativement différentiellement exprimées, dont 390 surexprimées chez les plérocercoïdes et 217 chez les adultes. L'annotation fonctionnelle Gene Ontology a révélé que les protéines surexprimées chez les plérocercoïdes étaient significativement enrichies en fonctions telles que le métabolisme des composés azotés, les complexes centraux du protéasome et la liaison aux ions. L'analyse d'enrichissement des voies métaboliques de la Kyoto Encyclopedia of Genes and Genomes (KEGG) a révélé une forte corrélation entre les protéines différentiellement exprimées (PDE) et les voies de transduction et de transport du signal, ainsi que les voies de catabolisme. De plus, l'analyse du réseau métabolique a mis en évidence l'implication des voies des pentoses phosphates et de la glycolyse/gluconéogenèse. Par ailleurs, un test ELISA indirect a montré que l'immunisation de souris avec des ESP induisait une réponse immunitaire mixte Th1/Th2, à prédominance Th1. La détection des cytokines a confirmé la bonne immunogénicité des ESP et leur capacité à activer les réponses immunitaires humorales et cellulaires. Cette étude révèle pour la première fois le profil d'expression différentielle des ESP entre les adultes et les plérocercoïdes de *S. mansoni*. Ces résultats offrent ainsi une piste potentielle pour le diagnostic et la prévention de la sparganose.

## Introduction

The zoonotic infection cestodiasis can seriously endanger human health and cause significant economic losses [27]. Currently, however, data for most groups of tapeworms in the order Diphyllobothriidea, such as *Spirometra mansoni*, are insufficient [30, 34]. Adults of *S. mansoni* mainly parasitize feline/canine animals, while the metacestode plerocercoids can parasitize humans and various vertebrates, causing a food/water-borne parasitic zoonosis known as sparganosis. It manifests mainly as larvae migrate, which may involve the whole body, resulting in blindness, limb paralysis, and even death [11, 37]. With a wide distribution, sparganosis has been reported in most countries worldwide [8, 12]. The clinical diagnosis of sparganosis relies mainly on biopsy of the parasite. Most patients usually have infections with only one or a few plerocercoids. It is challenging to observe the larval body, especially when the plerocercoid parasitizes the internal organs or the central nervous system [14]. Serological diagnosis is a preferable auxiliary diagnostic method, which, however, may lead to cross-reactivity and hence significantly compromised accuracy of diagnosis owing to a lack of specific antigens [33]. Prior research has documented the importance of the excretory–secretory proteins (ESPs) of parasites as ideal diagnostic antigens [20].

Tapeworm ESPs, produced by the parasite through excretion/secretion during infection, can be directly exposed to the host immune system, revealing an intimate association with the immune response of the host [32]. At present, relatively mature methods for obtaining tapeworm ESPs through *in vitro* culture have been developed, which have provided conditions for further study of the function of ESPs in the interplay between tapeworms and the host. Nono *et al.* reported that the eggs of *Echinococcus multilocularis* and the vesicles of the metacestode plerocercoid could inhibit dendritic cell maturation, thereby inducing antigen-specific immune tolerance and enabling *E. multilocularis* to evade immune activity [18, 19]. Rahimi *et al.* reported that the ESPs of protoscolices of *E. granulosus* could induce both Th1 and Th2 immune responses [24]. Pan *et al.* cocultured the ESPs of protoscolices of *E. granulosus* with mouse spleen CD19+ B cells, and verified via flow cytometry that it could regulate the negative immune response, allowing *E. granulosus* to evade immune activity [21, 22]. With respect to *Spirometra* tapeworms, the plerocercoid ESPs of *S. erinaceieuropaei* could inhibit the expression of tumor necrosis factor-α (TNF-α) in mouse peritoneal macrophages (Mφ) stimulated by lipopolysaccharide (LPS) or teichoic acid *in vitro* [4, 17]. Kina *et al.* reported that a glycoprotein in ESPs could inhibit osteoclastogenesis and the gene expression of proinflammatory cytokines [10]. Moreover, by stimulating mouse lymphocytes with *S. mansoni* ES, Kim *et al.* reported that it could suppress T lymphocyte proliferation and increase the negative regulation of Treg cells, which was beneficial for immune evasion by *S. mansoni* [9]. Despite some progress in ESP research on *S. mansoni*, there are currently no proteomics data on ESPs at different stages of its life cycle, which restricts research on the functional roles of ESPs in diagnosis, growth, and development, and host invasion mechanisms. In proteomics research, the 4D-Data Independent Acquisition (4D-DIA) method can achieve deep protein coverage, precise quantification, and low abundance protein detection through four-dimensional separation (retention time, m/z, intensity, and mobility), making it suitable for complex sample and/or trace sample research. In addition, this method effectively overcomes the biases and missing values of traditional DDA, and is more suitable for biomarker screening and dynamic protein change research [16, 35].

Specifically, in this study, the characteristics of ESPs of *S. mansoni* at different developmental stages were explored, and the results provide a foundation for further screening of candidate antigens and investigating their molecular functional mechanisms.

## Materials and methods

### Ethics approval

The study was conducted in accordance with the guidelines of the Declaration of Helsinki, and all procedures involving animals were approved by the Life Science Ethics Committee of Zhengzhou University (No. ZZUIRB GZR 2022-0142). The animals were handled in accordance with the good animal practices required by the Animal Ethics Procedures and Guidelines of the People’s Republic of China.

### Samples and experimental animals

**Figure 1:**
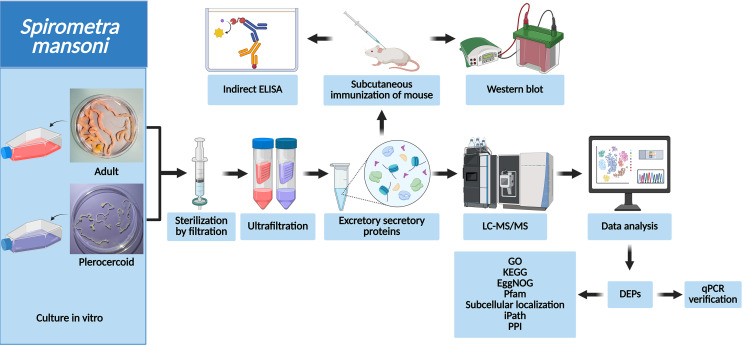
Flow chart of the entire study. Adults and plerocercoids were cultured *in vitro*. ES protein extraction, enzymatic hydrolysis, and enrichment were carried out. Peptide fragments were analyzed by LC–MS/MS. qRT–PCR quantitative verification and bioinformatics analysis of the differentially expressed proteins were performed.

Plerocercoids were isolated from a naturally infected snake (*Zaocys dhumnades* Cope, 1860), while adults were obtained from an infected cat according to previously described procedures [13]. Moreover, 4–6-week-old female BALB/c mice were purchased from the Henan Experimental Animal Center. Fresh plerocercoids and adults were incubated in medium, after which excretory–secretory proteins (ESPs) were collected. A flow chart of the procedure of the entire study is shown in Figure 1.

### Protein extraction, quantification, and peptide preparation

Plerocercoids (*n* = 3) and adults (*n* = 3) were sequentially washed with PBS and serum-free RPMI 1640 medium (both containing 100 U/mL penicillin and 100 μg/mL streptomycin). The organisms were then cultured *in vitro* in preheated, serum-free RPMI 1640 medium supplemented with antibiotics at 37 °C under 5% CO_2_, with the medium changed every 24 h. ESPs in the supernatant were collected, sterilized by 0.22 μm filtration, and concentrated in Millipore 10 kDa ultrafiltration centrifuge tubes (4,000× *g*, 4 °C, 40 min) to 500 μL, and stored at −80 °C until analysis. The protein concentration was quantified using the Bicinchoninic Acid Assay (BCA) method. For peptide preparation, 100 μg of protein was dissolved in 100 mM TEAB buffer, reduced with 10 mM TCEP (37 °C, 60 min), alkylated with 40 mM iodoacetamide (RT, 40 min, dark), and digested overnight at 37 °C with trypsin (1:50 w/w).

### DIA mass spectrometry detection

After trypsin digestion, the peptides were dried by vacuum pump. Afterwards, the enzymatically drained peptides were re-solubilized with 0.1% trifluoroacetic acid, then desalted with HLB, and dried by a vacuum concentrator. Afterwards, the peptides were quantified using a Pierce Quantitative Peptide Assays Kit (Thermo Fisher Scientific, Waltham, MA, USA). Furthermore, the quantified peptides were analyzed by a Vanquish Neo UHPLC coupled with an Orbitrap Astral mass spectrometer (Thermo Fisher Scientific) at Majorbio Bio-Pharm Technology Co. Ltd. (Shanghai, China). Briefly, the uPAC high-throughput column (75 μm × 5.5 cm, Thermo Fisher Scientific) was used with solvent A (water with 2% ACN and 0.1% formic acid) and solvent B (water with 80% ACN and 0.1% formic acid). The run time for chromatography was set to 8 min. Data-independent acquisition (DIA) data were acquired using an Orbitrap Astral mass spectrometer (Thermo Fisher Scientific) operated in DIA mode. The settings of the first-stage mass spectrum were as follows: voltage, 1.5 kV; scan range, 350–1300 m/z; resolution, 70,000; automatic gain control (AGC) target, 3 × 10^6^; and maximum injection time of the C-trap, 20 ms. The settings for the secondary mass spectrum were as follows: resolution, 17,500; AGC target, 5 × 10^5^; maximum injection time, auto; peptide fragmentation collision energy, 28%; variable window: 30; and total cycle time: 2.85 s.

### Protein annotations

On the basis of a search of the tapeworm proteome from UniProt, the proteomic data of proteomes closely related to that of *S. mansoni* with higher reliability were selected from the 9 reference proteomes for alignment. Given that high-quality reference proteomes for *S. mansoni* are lacking, the proteome of *Schistocephalus solidus*, which is closely related, was selected as an alternative. With the use of the *S. solidus* UP000275846 database, the search results were entered into Spectronaut to create a spectral library. Afterwards, daughter ion peak extraction and iRT correction were performed on the raw DIA data using Spectronaut™ 18/DIA-NN. Up to 6 specific peptide segments were selected for each protein, and three daughter ions were selected for each peptide for quantitative analysis. The following selection criteria were used: Protein FDR ≤ 0.01, peptide FDR ≤ 0.01, peptide confidence ≥ 99%, and XIC width ≤ 75 ppm. After the exclusion of shared and modified peptide segments, the sum of the peak areas was calculated to obtain quantitative results. The quantitative results of the identified proteins were subsequently standardized using total peak area comparison for DEP screening and statistical analysis. A list of qualitative and quantitative proteins in the sample was ultimately obtained for further use. In addition, to eliminate the effect of contaminating proteins on the results, common contaminating bases were added to the database.

### Screening of differentially expressed proteins (DEPs)

Pairwise comparisons between groups were performed using the LSD-*t* test, with the calculation of the significant *p*-value and fold change of differences between groups. In the three biological replicates, the fold change (FC) refers to the average expression ratio between ESPs of the adult and the plerocercoid. DEPs were proteins with *p* < 0.05 and FC > 1.5 or < 0.5. Additionally, a Venn diagram was generated to illustrate the relationships and differences in ESPs between adults and plerocercoids.

### Bioinformatics analyses

Full protein functional annotation analysis was conducted on the identified proteins using UniProt (https://www.uniprot.org). Gene Ontology (GO), Kyoto Encyclopedia of Genes and Genomes (KEGG) enrichment analysis, protein interaction network analysis, and iPath metabolic pathway analyses were performed on the DEPs. Classification annotation and cluster analysis were conducted on the basis of subcellular localization, molecular functions, and biological processes in the GO database (http://www.geneontology.org/) [7]. Annotation and enrichment analysis of these protein-related pathways were carried out through the KEGG database (https://www.genome.jp/kegg/). Moreover, functional annotation, enrichment, and subcellular localization analyses of the DEPs were completed using the EggNOG (http://eggnog5.embl.de/#/app/home), Pfam (http://pfam.xfam.org/), and subcellular localization databases. An overexpression analysis method was used for GO and KEGG enrichment analyses, with the implementation of a statistical significance test based on hypergeometric distribution. On the basis of a certain functional category annotation, this study calculated the *p*-value of the degree of significant enrichment of the experimentally identified or DEPs in a certain function, as well as the FDR correction value based on multiple hypothesis testing. The enrichment value was obtained by calculating the -log (*p*-value), and a corrected *p-*value of < 0.05 indicated significant enrichment. A higher enrichment value in the presence of a smaller *p-*value or FDR value, indicated obvious biological significance of the screened protein in the enriched function or pathway. To further explore the relationships between DEPs and protein interactions, a protein–protein interaction (PPI) network was constructed using the network modelling method from the online STRING database (http://string-db.org/) [3]. In this study, the topological properties of the network were analyzed, important PPI relationships were identified, and the results were visualized using Cytoscape software [26]. Eventually, the importance of each protein in the PPI network was evaluated by calculating its weight.

### Quantitative real-time PCR (qRT–PCR) for validation

Total RNA from *S. mansoni* adults and plerocercoids was extracted using the TRIzol method. In accordance with the instructions of the Novoprotein Reverse Transcription Kit, the qualified RNA obtained above was reverse transcribed into cDNA, which was stored at -80 °C for future use as a template for qRT–PCR. Afterwards, 10 proteins (5 upregulated and 5 downregulated) were randomly selected and confirmed at the transcriptional level using qRT–PCR. The primers used were synthesized by Sangon Biotech (Supplementary Table 1). GAPDH was used as the internal reference gene to normalize the same amplification reaction. In a reaction volume of 20 μL, 2 μL of 30-fold diluted cDNA was added as a template, with the addition of 10 μL of 2 × SYBR Green Pro Taq HS Premix (ROX plus), 0.4 μL (10 μM) of upstream and downstream primers, and 7.2 μL of ddH_2_O. qRT–PCR was performed in 96-well plates using an Applied Biosystems 7500 Fast Real Time PCR System (Applied Biosystems (Thermo Fisher Scientific, USA). The PCR conditions were as follows: initial pre-denaturation at 95 °C for 30 s, followed by 40 cycles of 95 °C for 30 s and 60 °C for 30 s. At the end of PCR amplification, melting curve analysis was performed from 60 °C to 95 °C at a gradual increase of 0.5 °C for 10 s. After amplification, the relative changes in differentially expressed genes were calculated using the 2^−ΔΔCt^ method. To ensure reproducibility of amplification, all qRT–PCR analyses of each gene were conducted with 3 biological replicates, with 3 replicates set for each sample. The significant differences between groups were analyzed using a *t*-test. A value of *p* < 0.05 indicated statistical significance. GraphPad Prism 10.1.2 was used for plotting.

### Functional analysis of the ESPs in *S. mansoni* plerocercoids

Twenty 8-week-old female BALB/c mice were randomly divided into five groups for immunization, with 4 mice in each group. The antigen was mixed with an equal volume of the adjuvant at a 1:1 ratio and injected subcutaneously into the mice at multiple locations on the 1st, 14th, and 28th days, with a dosage of 20 μg/mouse for each antigen. Freund’s complete adjuvant was used for the first immunization, whereas Freund’s incomplete adjuvant was used for the second and third immunizations. Tail vein blood was collected from the mice in each group prior to the first immunization, 7 days after the first immunization, and 7 days after the second immunization. Eye blood was collected from the mice two weeks after the third immunization. The collected blood samples were placed at 37 °C for 2 h and centrifuged at 3,000× *g* for 15 min. Then, the clear serum in the upper layer was divided into aliquots and stored at −80 °C. ES and soluble proteins of the worms were subsequently identified using western blotting. The primary antibody was the mouse serum after three immunizations (1:100), and the secondary antibody was HRP-labelled rabbit anti-mouse IgG (1:5,000). Samples (15 μg protein) mixed with 5× loading buffer were boiled for 10 min and separated by SDS–PAGE (80 V stacking gel, 120 V separating gel) until the bromophenol blue reached the bottom. The proteins were wet-transferred to pre-activated PVDF membranes (0.22 μm) at 200 mA for 1–2 h in transfer buffer (25 mM Tris, 0.2 M glycine, 20% methanol). The membranes were blocked with 5% non-fat milk in TBS-T for 1 h at 37 °C and then incubated with primary antibodies overnight at 4 °C. After being washed (3 × 5 min in TBS-T), the membranes were incubated with HRP-conjugated secondary antibodies (1:5,000) for 1 h at 37 °C. The protein bands were visualized by chemiluminescence (ECL A/B mix) and detected using an imaging system. Indirect ELISA was used to measure the specific total levels of IgG, IgG1, IgG2a, and IgM against the corresponding antigens in the serum of each group. In addition, the serum levels of the cytokines IL-4, IL-10, IL-12, and IFN-γ in the serum of each group were assessed using corresponding ELISA kits.

### Data deposition

All the data supporting the findings of this study are included in the main article and its supplementary files. The mass spectrometry proteomics data have been deposited in the ProteomeXchange Consortium (http://proteomecentral.proteomexchange.org) via the iProX partner repository in the PXD065541 dataset.

## Results

### DIA-based quantitative proteomic analysis of ESPs

**Figure 2:**
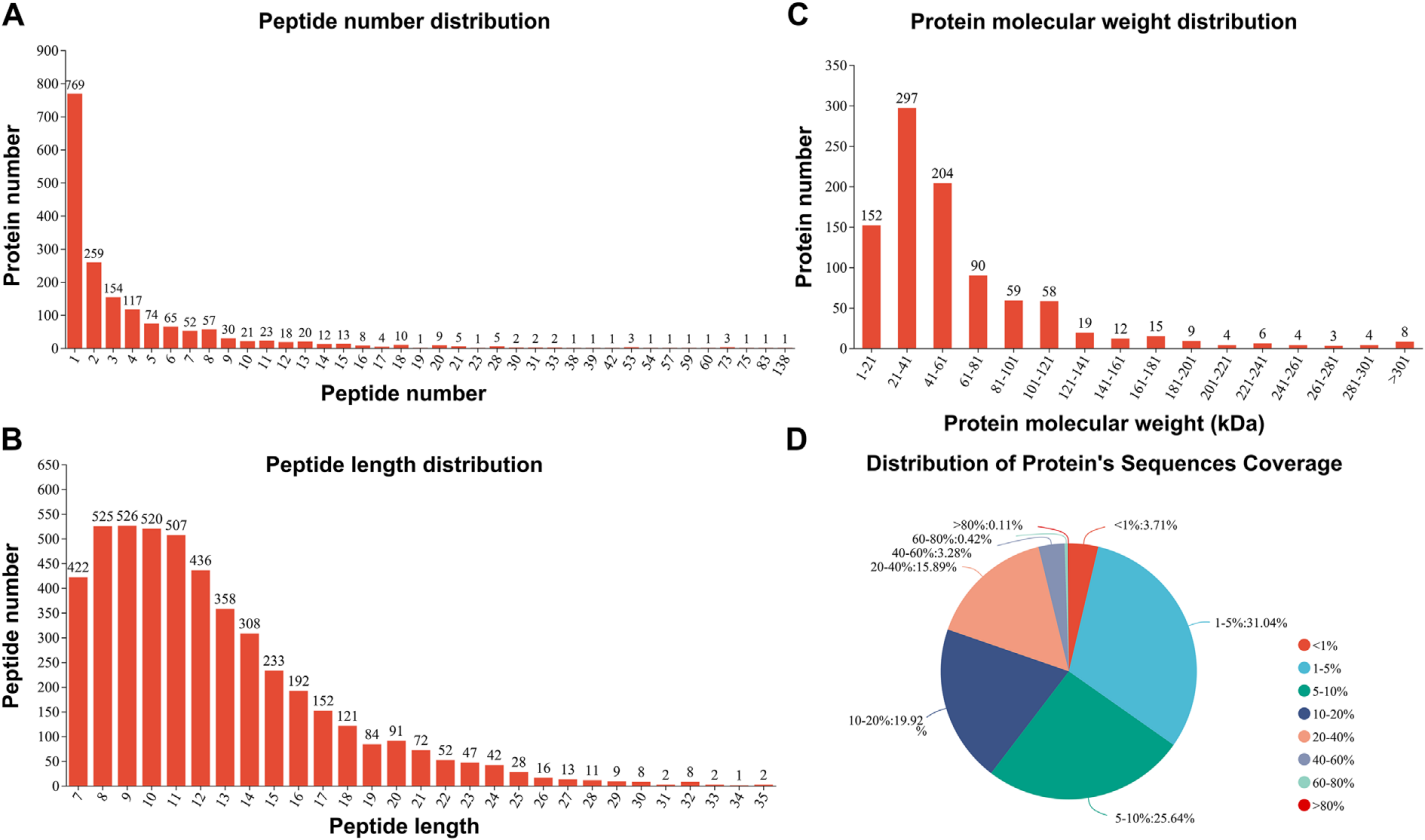
Identification of peptide segments and proteins in the ESP of *Spirometra mansoni* by 4D DIA analysis. (A) Peptide number distribution. (B) Peptide length distribution. (C) Protein molecular weight distribution. (D) Protein coverage distribution.

According to peptide diversity analysis, a total of 4,908 peptide segments were identified in 6 samples (3 plerocercoids and 3 adults), with 769, 604, 225, and 151 proteins containing single peptides, 2–5 peptides, 6–10 peptides, and ≥11 peptides, respectively (Fig. 2A). The identified protein peptides were concentrated in 7–14 peptide segments, with a maximum of 35 peptide segments (Fig. 2B). Afterwards, 944 proteins were identified through comparison of the 4,908 peptide segments to those in the UniProt database, with molecular weights concentrated mostly between 1 and 121 kDa (Fig. 2C). In addition, only 186 proteins (19.7%) had known amino acid sequences accounting for > 40% (Fig. 2D).

**Figure S1.**
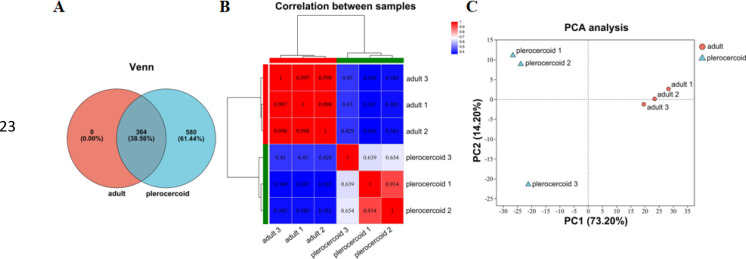
Comparative analysis between ESP samples. (A) Venn diagram of protein sets identified in adult and plerocercoid. (B) Correlation analysis of six samples. (C) PCA analysis of six samples.

Quantitative analysis was conducted on the identified proteins, with 364 and 944 proteins identified in adults and plerocercoids, respectively, as shown in the Venn diagram. Among them, 580 proteins were specifically expressed in the plerocercoid stage; however, adult-specific proteins were not observed (Supplementary Fig. S1A). There was a strong correlation between each sample, suggesting good repeatability (Supplementary Fig. S1B). Principal component analysis (PCA) revealed relatively low sample variability among the three biological replicates, with significant differences between the adult and plerocercoid groups (Supplementary Fig. S1C).

**Figure 3:**
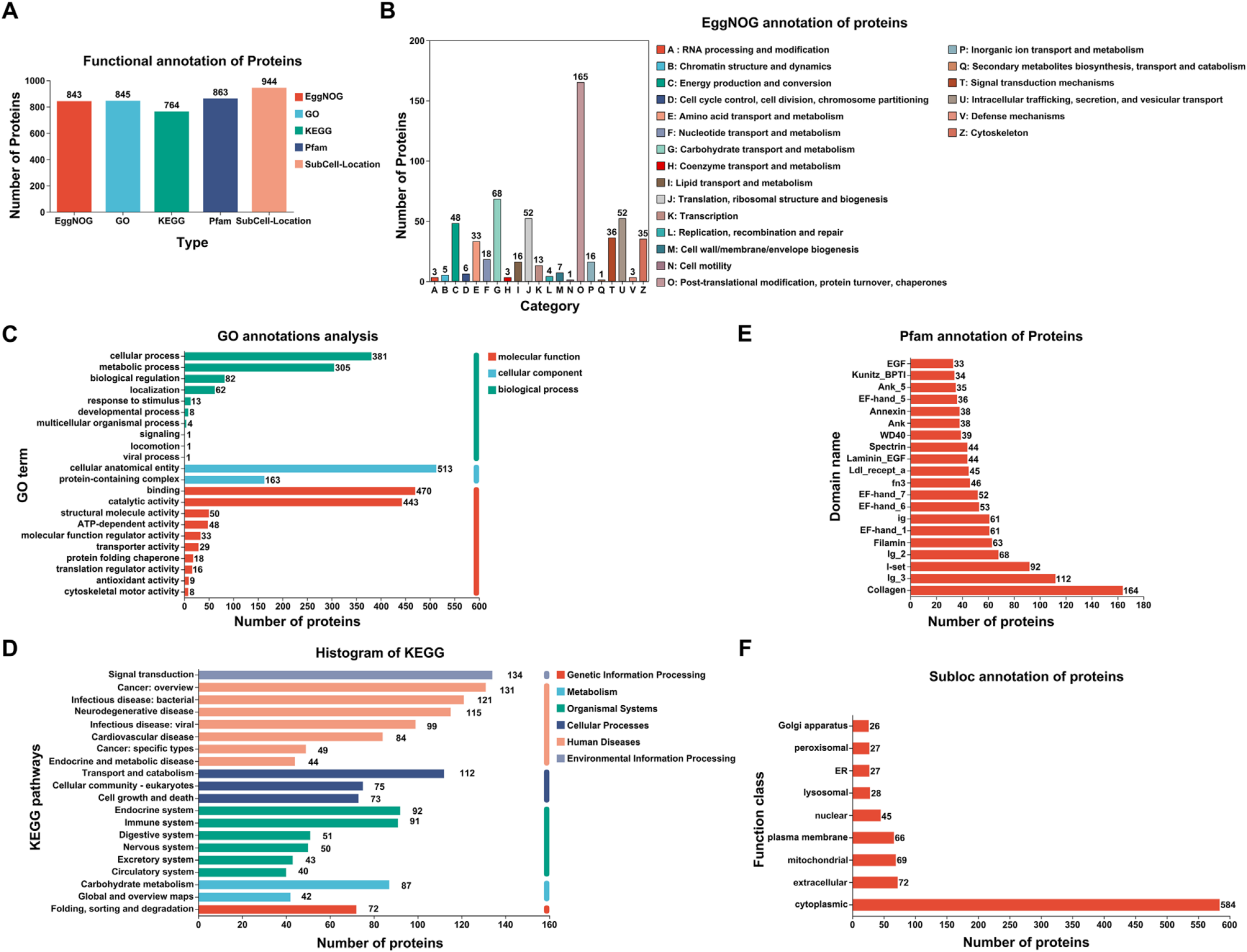
Annotation of ESPs in *Spirometra mansoni*. (A) Bar charts of the annotation results for different databases. (B) EggNOG annotation. (C) Statistical bar chart for GO classification. (D) Statistical chart for important enriched pathways identified by KEGG analysis. (E) Bar chart for Pfam annotation. (F) Statistical bar chart for subcellular localization.

**Figure S2.**
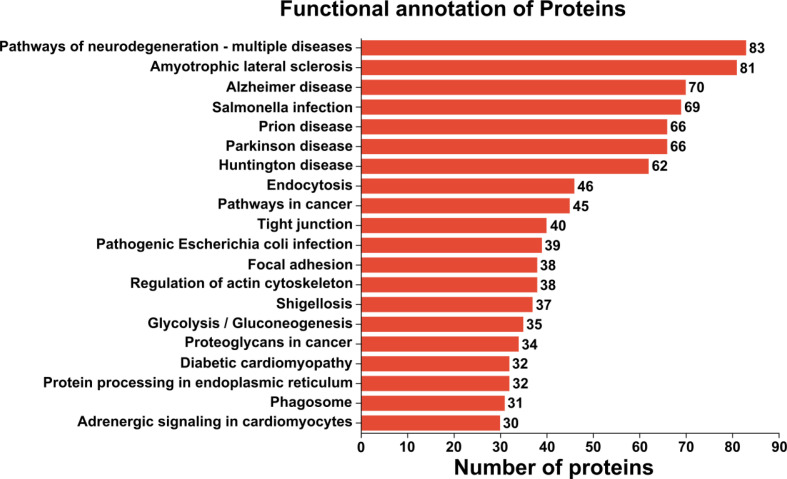
Statistical bar chart for pathway classification of ESPs in *S. mansoni*.

Of the 944 identified proteins, 843 could be annotated in EggNOG, 845 could be annotated in the GO database, 764 could be annotated in the KEGG database, and 863 could be annotated in the Pfam; in addition, all the proteins could be annotated in the subcellular localization database (Fig. 3A). In terms of the EggNOG database, primary annotations were found for posttranslational modification, protein turnover, and chaperones (Fig. 3B). On the basis of the results of the GO analysis, 381 protein annotations were in cellular process in the biological process (BP) category, 513 were in cellular anatomical entity in the cellular component (CC) category, and 470 were binding in the molecular function (MF) category (Fig. 3C). The most annotated pathways in the KEGG were signal transduction, cancer overview, and transport and catabolism (Fig. 3D). Pfam analysis revealed that these protein domains were mainly annotated in collagen, Ig_3, and I-set (Fig. 3E). Subcellular localization statistical analysis revealed that most annotated proteins were located in the cytoplasm (Fig. 3F). Further statistical analysis of important pathways revealed 83 proteins associated with pathways related to neurodegeneration and multiple diseases (Supplementary Fig. S2).

**Figure 4:**
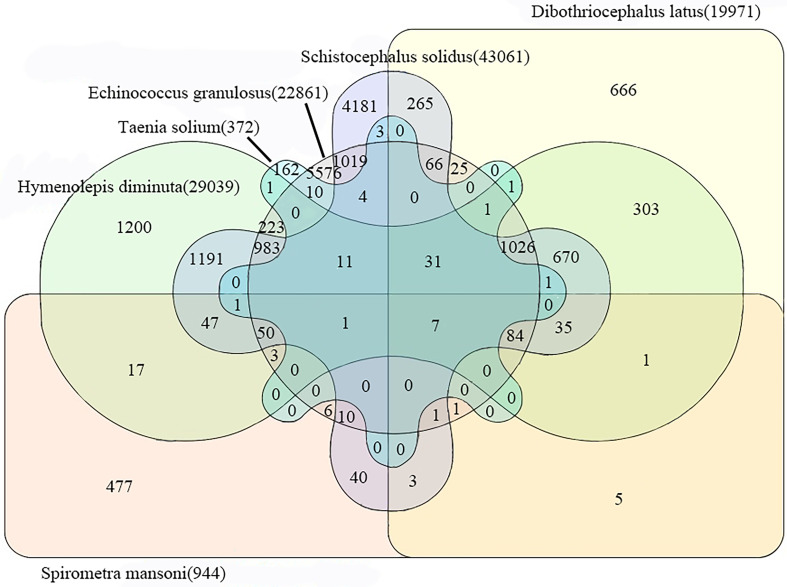
Comparison of the proteomes of the ESPs of *Spirometra mansoni* and five closely related tapeworms using a Venn diagram.

The proteomic similarity between the ESPs of *S. mansoni* and those of five other closely related tapeworms (*Taenia solium*, *Echinococcus granulosus*, *Hymenolepis diminuta*, *Dibothriocephalus latus*, and *Schistocephalus solidus*) was visualized through a Venn diagram, as shown in Figure 4. Consequently, 467 proteins in the ESPs of *S. mansoni* shared similarities with those of five types of tapeworms. Specifically, the proteins Wnt, calreticulin, paramyosin, CS domain-containing protein, dynein light chain, annexin, and tetraspanin were common in all the proteomes. These proteins could interact to form functional networks and participate in core biological processes such as cell signal transduction, structural maintenance, and movement regulation, functioning significantly in pathological and physiological processes such as growth and development, immune response, and tumorigenesis.

### Screening and identification of DEPs

**Figure 5:**
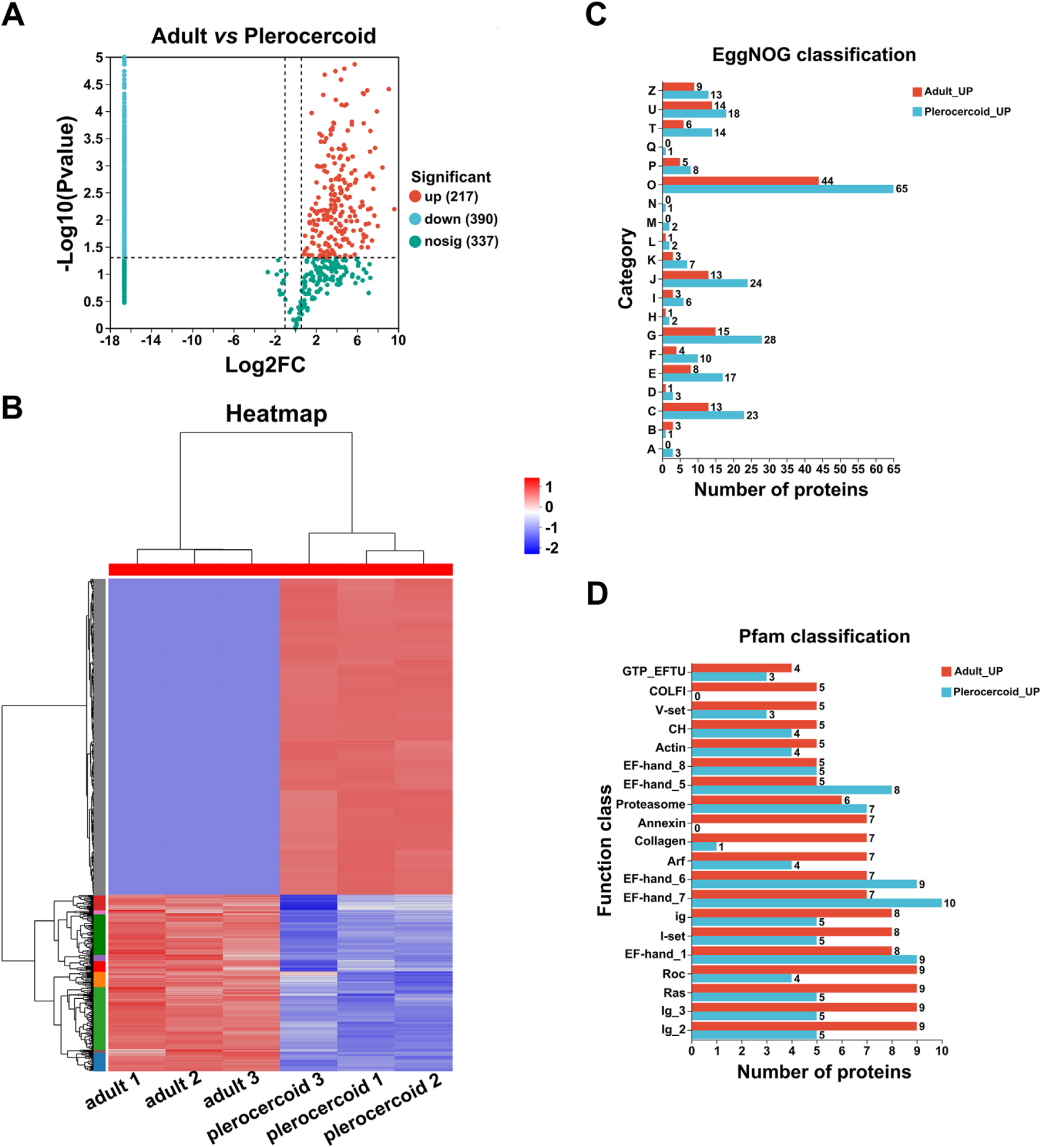
Screening of ESPs differentially expressed between adults and plerocercoids. (A) Volcano map of the DEPs. (B) Hierarchical clustering heatmap of the DEPs. (C) EggNOG analysis of upregulated proteins in adults and plerocercoids. (D) Pfam analysis of upregulated proteins in adults and plerocercoids.

In accordance with the results of the quantitative analysis on the expression levels of 944 proteins, there were 607 statistically significant DEPs (*p* < 0.05 and FC > 1.5 or < 0.5), among them, 217 were upregulated in adults and 390 were upregulated in plerocercoids (Figs. 5A, 5B).

### EggNOG, Pfam, and subcellular localization analyses of DEPs

**Figure S3.**
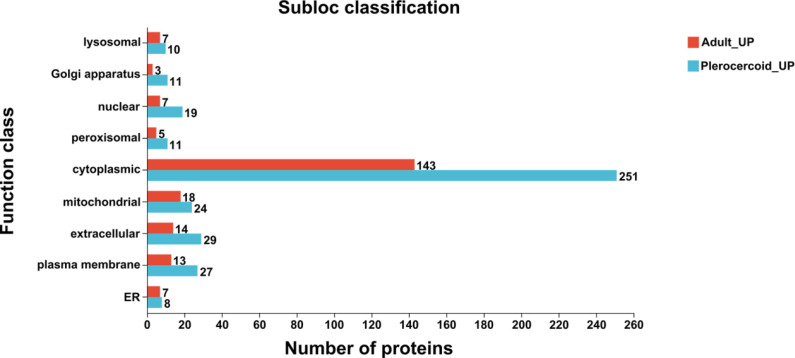
Subcellular localization analysis of up-regulated proteins in adult and plerocercoid.

A comparison of the upregulated proteins in adults and plerocercoids with those in the EggNOG database revealed that most of these proteins were involved in posttranslational modifications, protein conversion, and chaperoning (Fig. 5C). When compared with data from the Pfam database, the domains with higher annotation levels in the upregulated proteins in adults were Roc, Ras, Ig_2, and Ig_3, whereas the upregulated proteins in the plerocercoids were annotated more frequently as EF-hand-1, EF-hand-7, and EF-hand-6 domains (Fig. 5D). EF-hand domains, as calcium sensors, are prevalent in helminth ESPs and regulate calcium signalling to mediate host–parasite interactions, including immune evasion, tissue invasion, and nutrient acquisition; their upregulation suggests crucial roles in sparganum infection. Moreover, according to the subcellular localization database, the cytoplasm was the major location for the majority of upregulated proteins in adults and plerocercoids (Supplementary Fig. S3).

### GO analysis of DEPs

**Figure 6:**
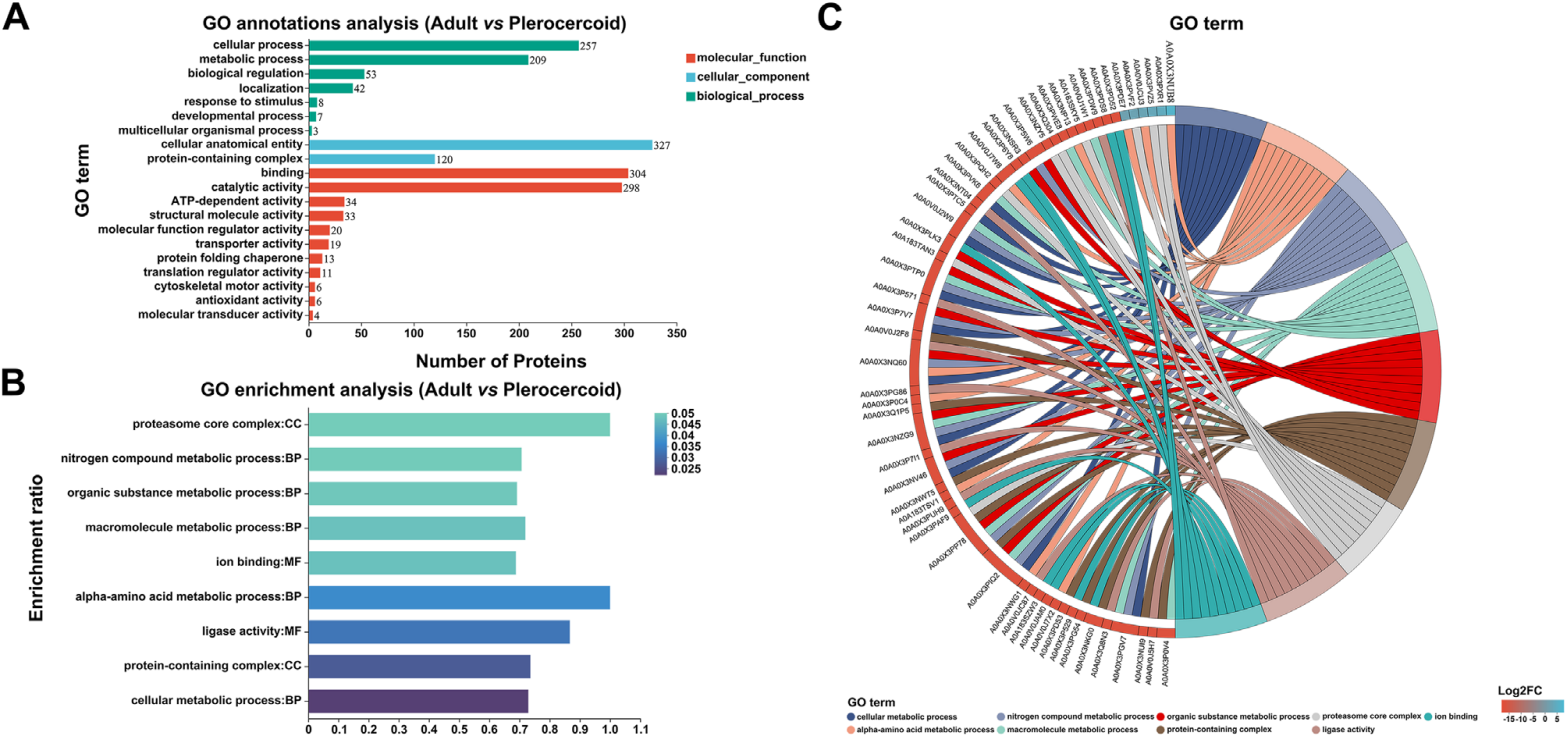
GO classification statistical analysis and enrichment analysis of DEPs (*p* < 0.05). (A) Statistical bar chart of the GO classifications of the differentially expressed proteins. (B) Bar chart of the GO enrichment analysis of the differentially expressed proteins. (C) DEP GO enrichment string plot.

**Figure S4.**
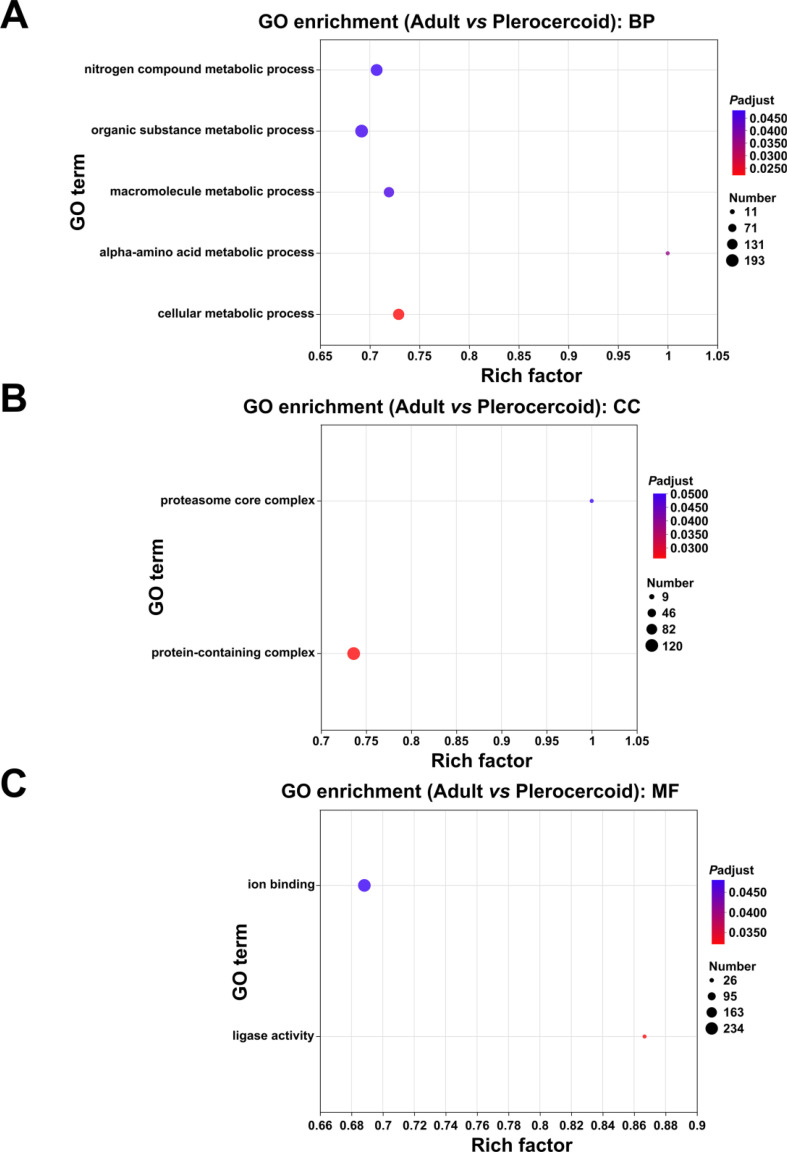
GO enrichment analysis of DEPs.

**Figure S5.**
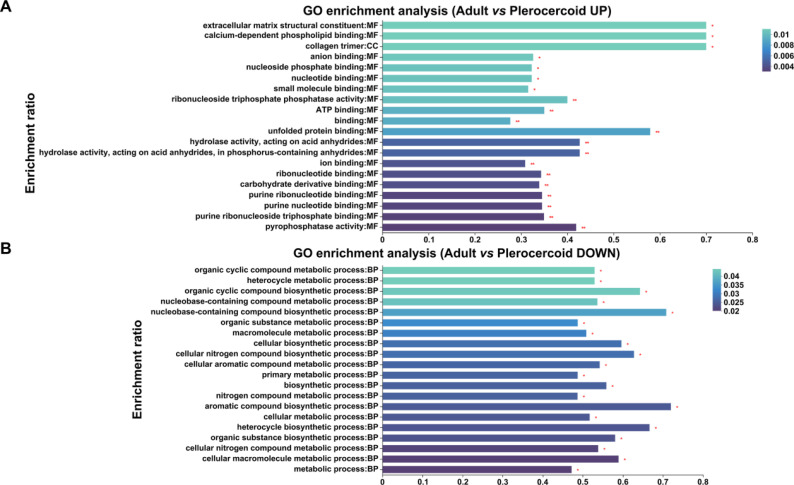
GO enrichment analysis of upregulated DEPs.

The 607 DEPs with statistical significance were annotated by 26 GO terms, including 581 related to BP (mostly related to nitrogen compound metabolism mostly), 447 related to CC (primarily related to proteasome core complexes primarily), and 753 related to MF (generally related to ion binding generally) (Figs. 6A and 6B, Supplementary Fig. S4). In addition, protein enrichment by STRING could connected different proteins with their corresponding GO terms, which could facilitate an intuitive identification of proteins with significantly higher differential expression levels in significantly enriched GO terms (Fig. 6C), including A0A0X3PVK6 in cellular metabolism and A0A0X3NUB8 in alpha amino acid metabolism. Further GO enrichment analysis revealed that 217 upregulated proteins in adults were annotated by 23 GO terms, and 390 upregulated proteins in plerocercoids were annotated by 24 GO terms. Specifically, the highly enriched terms in adults included extracellular matrix structural component, calcium-dependent phospholipid binding, and collagen trimer (Supplementary Fig. S5A); whereas those in plerocercoids included the biosynthesis processes of aromatic compounds, nucleoside compounds, and heterocyclic compounds (Supplementary Fig. S5B).

### KEGG analysis of DEPs

**Figure 7:**
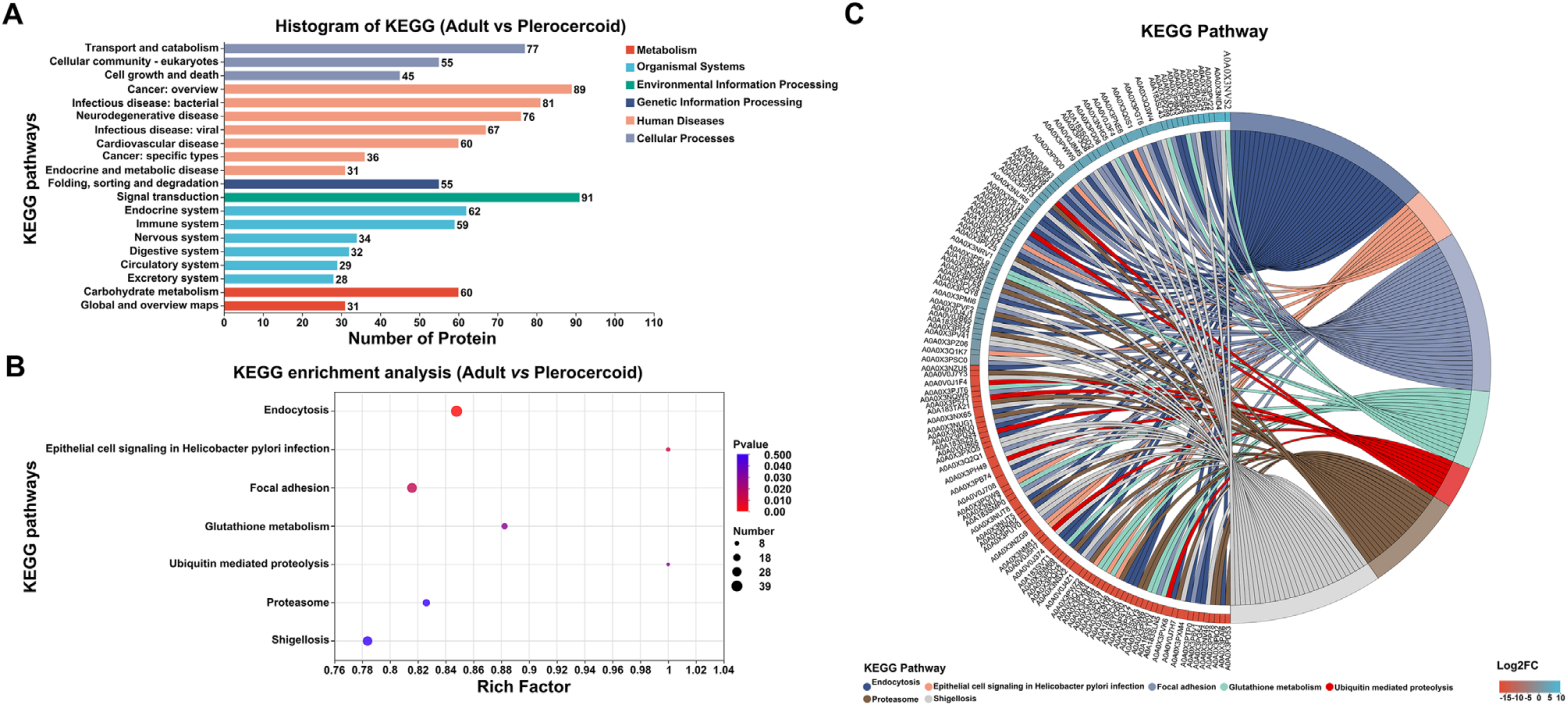
KEGG pathway analysis of ESPs differentially expressed between adults and plerocercoids (*p* < 0.05). (A) Statistical bar chart of the KEGG classification of the DEPs. (B) KEGG enrichment of pathways of the DEPs. (C) KEGG pathway enrichment by STRING of upregulated DEPs.

**Figure S6.**
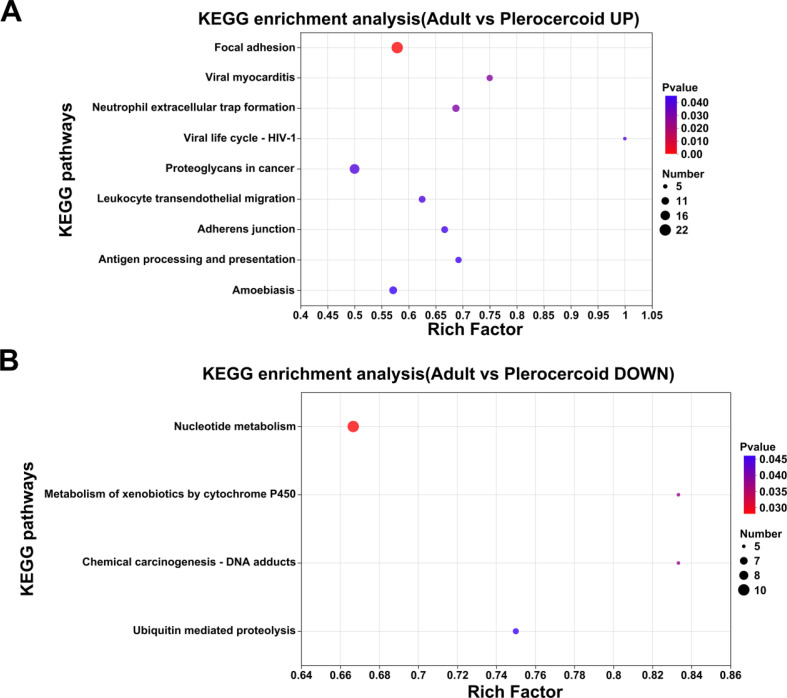
KEGG pathway enrichment entries of upregulated DEPs.

When the DEPs were compared with those in the KEGG database, the DEPs were found to be involved in six out of the seven KEGG pathways (Fig. 7A). The significantly enriched pathways included endocytosis, focal adhesion, glutathione metabolism, and ubiquitin mediated proteolysis (Fig. 7B, Supplementary Fig. S6). As indicated by the STRING enrichment, specific proteins with significantly higher differential expression in the KEGG pathway included A0A0V0JA57 in endocytosis and A0A0X3NID4 in adhesive plaques, etc. (Fig. 7C).

### iPath metabolic pathway analysis of DEPs

**Figure 8:**
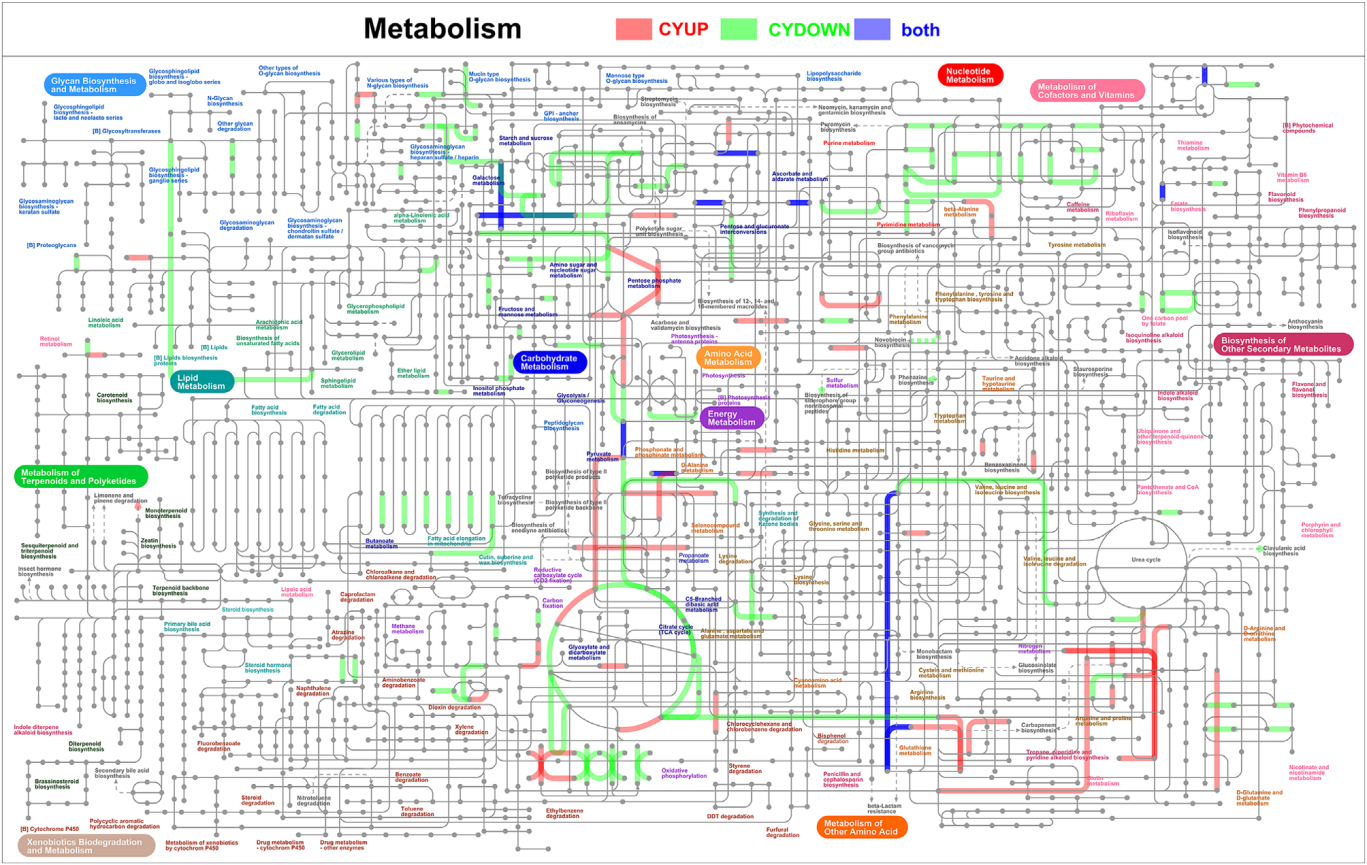
Map of the iPath integration path diagram of the metabolic pathway.

**Figure S7.**
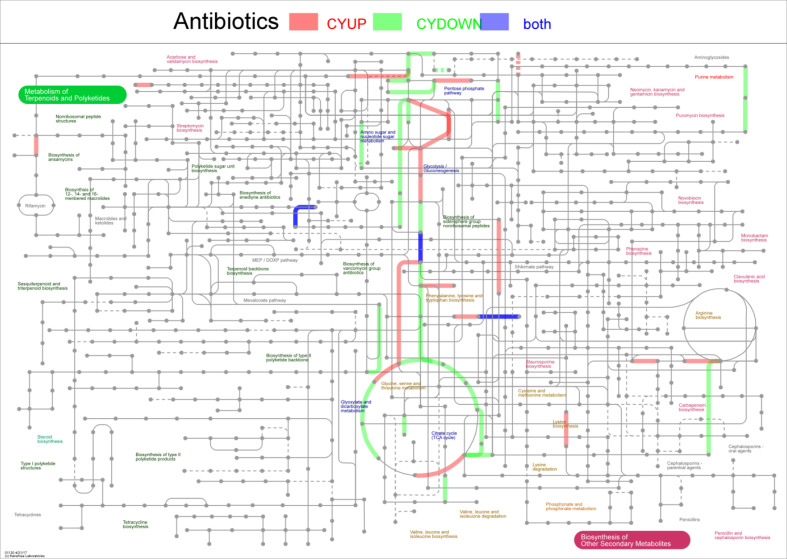
Map of the Ipath integration path diagram of biosynthesis of antibiotics.

**Figure S8.**
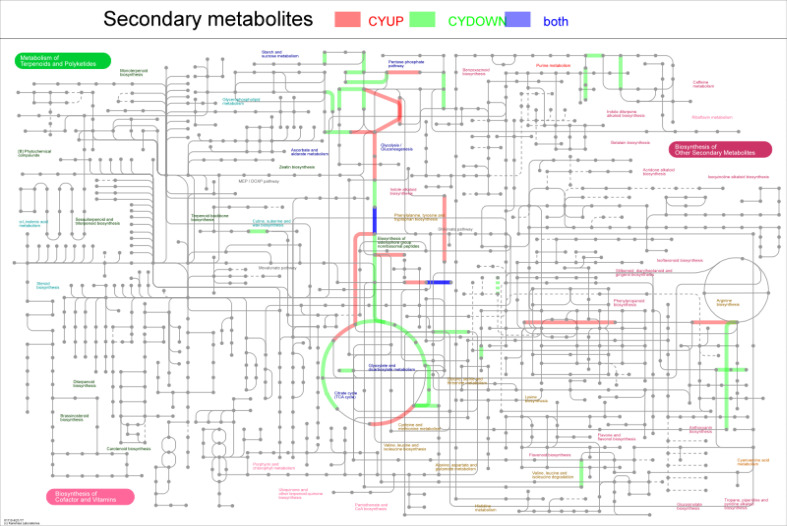
Map of the Ipath integration path diagram of biosynthesis of secondary metabolites.

**Figure S9.**
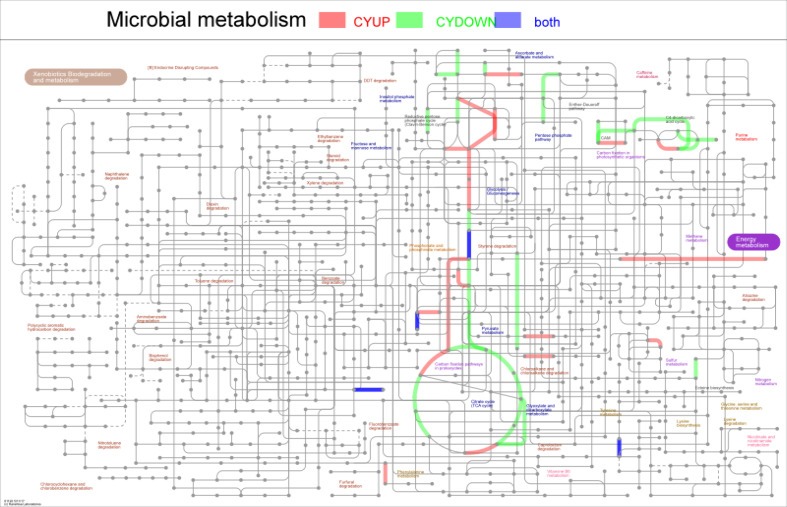
Map of the Ipath integration path diagram of microbial metabolism in diverse environments.

The metabolic pathways were visualized to obtain information on the metabolic pathways of the entire biological system. A complete overview of metabolic pathways is shown in Figure 8, while complete overviews of antibiotic biosynthesis, secondary metabolite biosynthesis, and microbial metabolism in different environments are presented in Supplementary Figs. S7–S9, respectively. The most significant metabolic pathways included the pentose phosphate pathway, amino and nucleotide sugar metabolism, glycolysis/gluconeogenesis, glyoxylate and dicarboxylate metabolism, and the citric acid cycle.

### PPI analysis of DEPs

**Figure 9:**
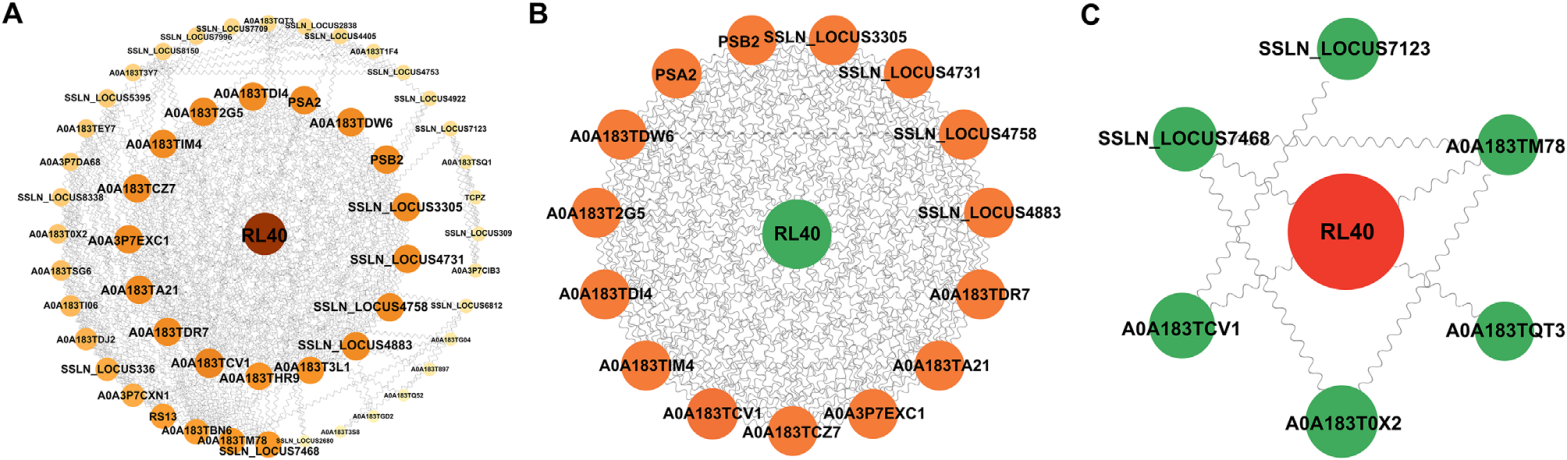
PPI network of differentially expressed ESPs in *Spirometra mansoni*. (A) The PPI network of all 43 DEPs. (B) The PPI network of proteins with a higher degree. (C) The PPI network of proteins with high betweenness centrality scores.

Using the STRING database, a PPI network (Fig. 9A) was constructed for the 43 DEPs that were significantly enriched in both the GO and KEGG databases. RL40 stood out and interacted closely with the other proteins. Further screening of proteins with extensive connections in the network revealed 16 nodes with ≥17 connections, among which RL40 remained at the core node position (Fig. 9B). Moreover, analysis of the DEPs connecting different modules in the network indicated that RL40 was closely related to other key nodes, suggesting that RL40 plays a pivotal role in the PPI network (Fig. 9C).

### Validation of DEPs

**Figure 10:**
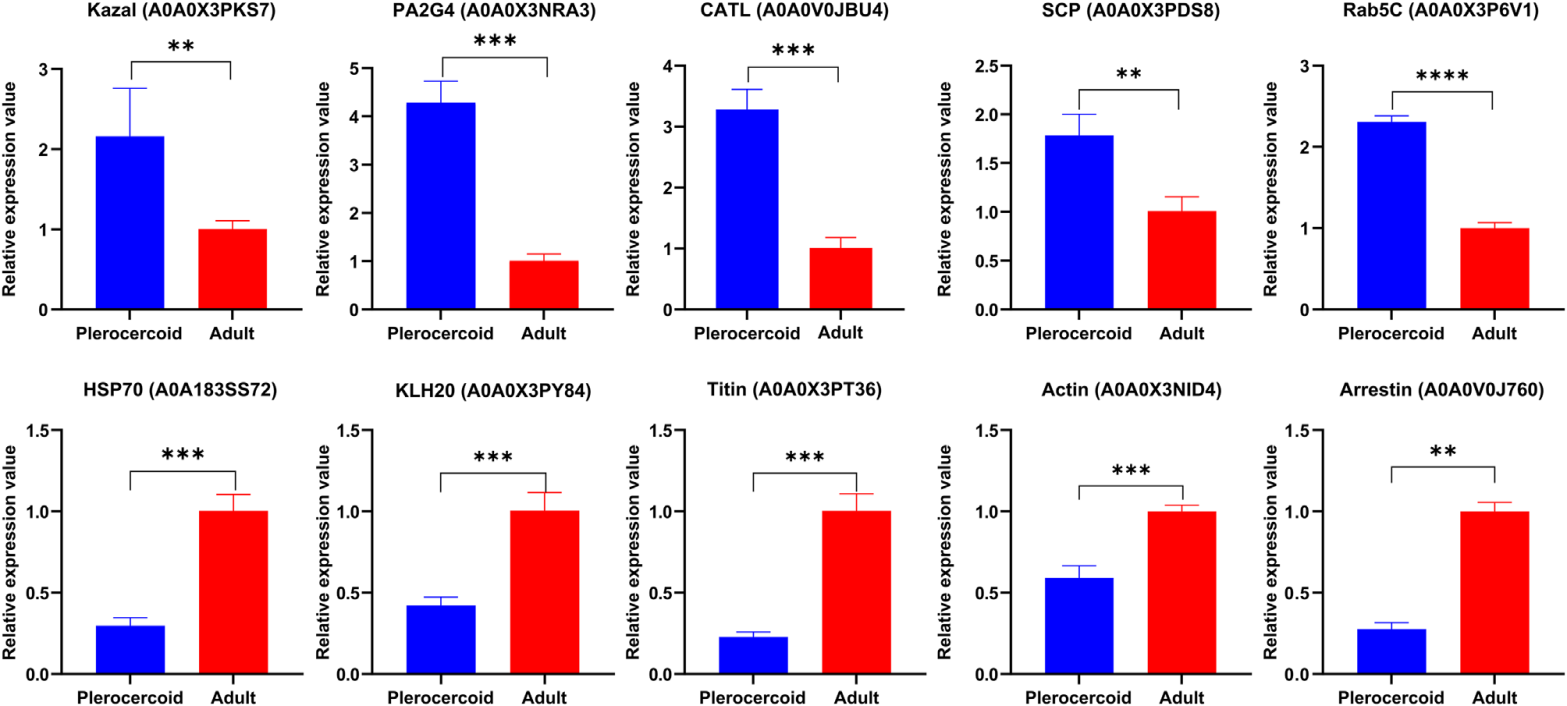
qRT–PCR validation of differentially expressed genes of ESPs in *Spirometra mansoni*. GAPDH was used for normalization. The results are given as the mean±SEM (standard mean of error) of the samples (*n* = 3). Asterisks indicate significant differences (*p < 0.05, **p < 0.01, ***p < 0.001, and ****p < 0.0001).

To verify the accuracy of ES proteomics data, 5 upregulated proteins in adults and 5 upregulated proteins in plerocercoids were randomly selected for qRT–PCR analysis (Fig. 10). The 5 proteins from the plerocercoid group were A0A0X3PKS7 (Kazal, *t*_Kazal_ = –5.583, *p* < 0.05); A0A0X3NRA3 (PA2G4, *t*_PA2G4_ = 12.129, *p* < 0.001); A0A0V0JBU4 (CATL, *t*_CATL_ = 10.584, *p* < 0.001); A0A0X3PDS8 (SCP, *t*_SCP_ = 5.113, *p* < 0.01) and A0A0X3P6V1 (Rab5C, *t*_Rab5C_ = 22.537, *p* < 0.0001); while the five proteins from the adult group were A0A183SS72 (HSP70, t_HSP70_ = –10.857, *p* < 0.001); A0A0X3PY84 (KLH20, t_KLH20_ = –8.203, *p* < 0.05); A0A0X3PT36 (Titin, *t*_Titin_ = −12.319, *p* < 0.05); A0A0X3NID4 (actin, *t*_Actin_ = –8.449, *p* < 0.05); and A0A0V0J760 (arrestin, *t*_Arrestin_ = –5.583, *p* < 0.05). All these proteins exhibited upregulated expression, which was consistent with the omics sequencing, confirming the accuracy and reliability of the sequencing results.

### Immunoassay of the plerocercoid ESPs

**Figure 11:**
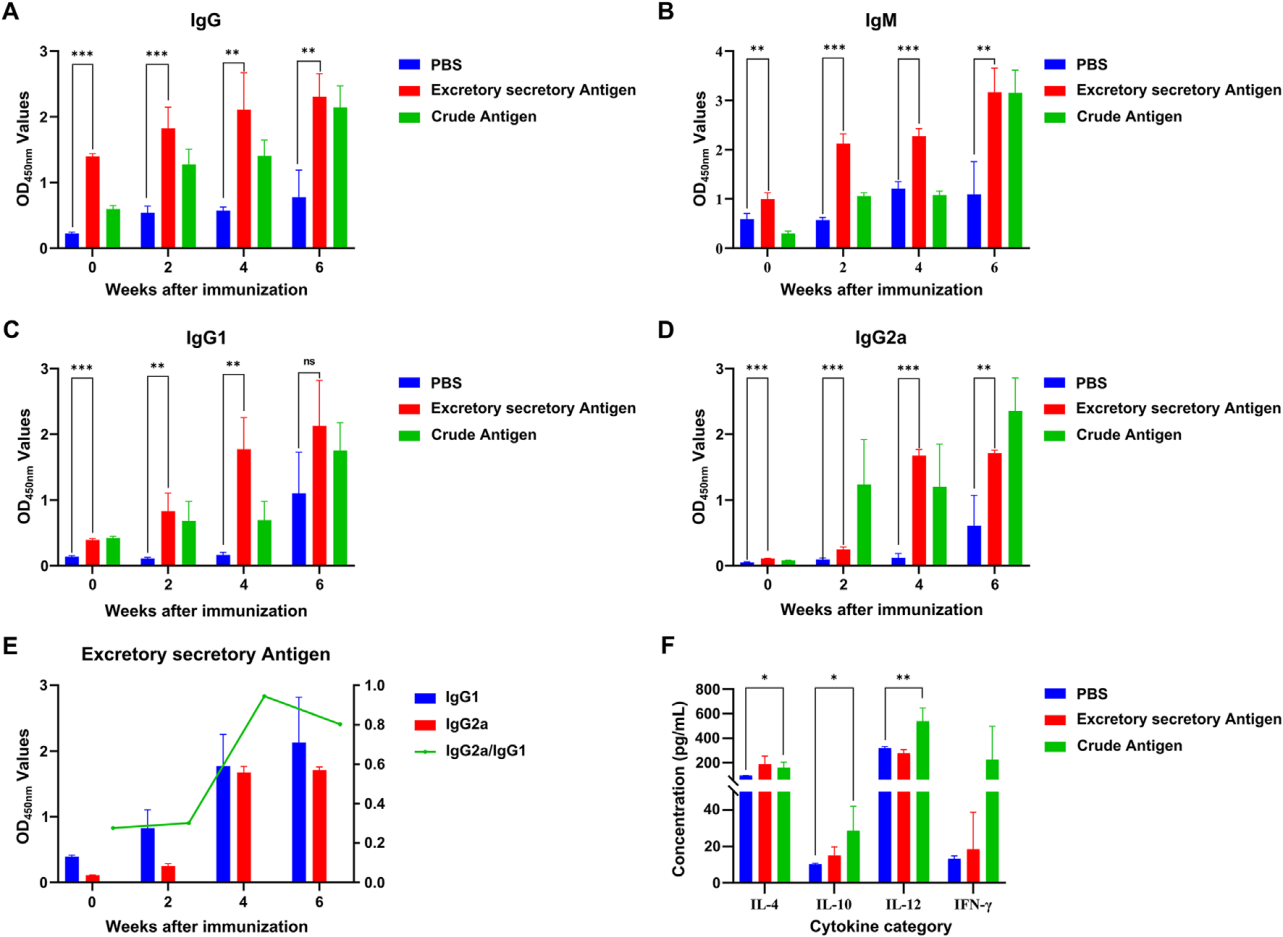
Determination of antibody levels in plerocercoids. (A) IgG antibody level. (B) IgM antibody level. (C) IgG1 antibody level. (D) IgG2a antibody level. (E) Comparison of serum IgG1 and IgG2a antibody levels in ESP-immunized mice. (F) Cytokine determination.

According to the detection of IgG, IgM, IgG1, and IgG2a antibody levels by indirect ELISA in ESP-immunized mice, the IgG antibody level significantly increased in the ESP immunization group compared with that in the PBS and crude antigen groups, with significant differences (both *p* < 0.05), indicating that ESPs could induce humoral immune responses in mice (Fig. 11A). IgM levels increased after the first immunization, but the rate of increase slowed down after the second immunization and reached its highest level after the third immunization, indicating that ESPs could induce the production of high IgM antibody levels (Fig. 11B). Furthermore, after the first immunization, the IgG1 antibody level rapidly increased in the ESP immunization group, peaking at the third immunization (Fig. 11C). The IgG2a antibody level increased rapidly and significantly differed from that in the control group (*p* < 0.001) but decreased slightly after the third immunization (Fig. 11D). In addition, the IgG2a/IgG1 ratio was significantly greater in the ESP immunization group after the third immunization than that in the PBS and crude antigen groups, indicating that ESPs might induce a Th1-biased Th1/Th2 mixed immune response in mice (Fig. 11E). Moreover, there were significant differences in the serum IL-4, IL-10, and IL-12 levels of the mice before and after the three immunizations in the ESP immunization group (all *p* < 0.05), and these differences were accompanied by significantly increased IFN-γ levels, indicating that ESP could induce cellular immune responses in mice (Fig. 11F). Compared with that in the control group, the increase in IFN-γ levels in the serum of mice in the ESP immunization group after three immunizations was significantly greater than that of IL-4. These results indicate that the ESPs induced a Th1-biased Th1/Th2 mixed immune response in mice on the basis of changes in the IgG2a/IgG1 ratio.

## Discussion

In this study, the expression profiles and functional characteristics of ESPs in *S. mansoni* plerocercoids and adults were systematically analysed using DIA-based quantitative proteomics technology. Our study revealed 944 proteins, of which 580 were specifically expressed in the plerocercoid, but no specific proteins were found in the adults. The high expression level of plerocercoid-specific proteins was consistent with the findings in *E. granulosus*, in which the diversity of secreted proteins during the larval stage was reported to be strongly related to their invasion of host tissues and immune escape strategies [15]. However, the lack of specific proteins expressed during the adult stage was in contrast to that in schistosomiasis, in which multiple adult-specific reproductive-related secretory proteins were identified [29]. This difference may be attributed to distinct biological characteristics among tapeworm species, as adult tapeworms rely on host-derived nutrient absorption, which may lead to redundant protein secretion. Additionally, variations in the host environment could influence the patterns of protein secretion. Moreover, owing to limitations in the sample, the present study was not able to fully examine the constraints associated with host-specific effects. Our functional enrichment analysis revealed that the 944 proteins identified above were involved mainly in posttranslational modifications, protein conversion, and carbohydrate metabolism, *etc*. The enrichment of posttranslational modifications (*e.g.*, ubiquitination) and carbohydrate metabolism pathways is consistent with the general pattern of energy metabolism remodelling during parasite development [25]. For example, the significant enrichment of partner proteins (*e.g.*, the HSP family) may facilitate the maintenance of protein homeostasis to assist in coping with host environmental stress [1]. Further KEGG analysis revealed that ESPs were significantly enriched in pathways such as signal transduction and neurodegenerative diseases. Notably, there is currently insufficient research on the significant enrichment of pathways related to neurodegenerative diseases (*e.g.*, Alzheimer’s disease and amyotrophic lateral sclerosis), and in studies of other parasitic ESPs. As a result, on the basis of the current evidence, plerocercoids may interfere with the host immune system possibly by mimicking host pathologic pathways, such as abnormal protein aggregation or oxidative stress responses, which requires further investigation of the specific mechanism involved. Comparative proteomic analysis identified 607 DEPs. The upregulated proteins in adults were significantly enriched in pathways involved in functions such as focal adhesion, adherens junction formation, and antigen processing and presentation. These proteins serve as signals that regulate cell adhesion, mechanical sensing, and cell junctions and control cell growth and differentiation, which may be closely associated with the parasitism of adults in the host gut. Moreover, the proteins upregulated in plerocercoids were significantly enriched in nucleotide metabolism, metabolism of xenobiotics by cytochrome P450, and ubiquitin-mediated proteolysis pathways, occupying a critical position in regulating the synthesis, breakdown, and metabolism of nucleic acids and proteins. Furthermore, PPI analysis revealed several key node proteins, among which RL40 stood out and interacted closely with other key nodes. As a key component of large ribosomal subunits, RL40 can mediate protein synthesis and ubiquitination modification through its conserved ubiquitin domain, and regulate abnormal protein degradation and cell cycle progression, thereby contributing significantly to the occurrence of cancer (*e.g.*, breast cancer and lung cancer), nervous system diseases and cardiovascular diseases [23, 31]. Therefore, the RL40 of *S. mansoni* may play an important role in information transmission or signal transduction by synergistically regulating protein synthesis and degradation through the dual function of the ribosome and ubiquitin system. In addition, some plerocercoid-specific proteins (*e.g.*, ATG7 and actin-binding protein) were also observed to have potential as diagnostic markers or vaccine targets.

Research on the immune function of ESPs has revealed that multiple antigen bands can be recognized by the immune serum against the excretory–secretion antigens from the plerocercoids, whereas low-molecular-weight (< 20 kDa) antigens are not recognized by infected serum. In this context, owing to insufficient secretion or immune escape mechanisms, ES antigens may not be effectively recognized in natural infections. In contrast, the recognition results of the soluble proteins in the immune serum of the parasite were consistent with those of the infected serum, indicating unique immunogenicity possessed by the ES antigens. In our study, on the basis of the ELISA results, ESPs induced a significant increase in IgG and IgM antibody levels, with a continuous increase in the levels of specific IgG1 and IgG2a antibodies as the number of immunizations increased. These dynamic changes indicate that ESPs can activate Th1/Th2 mixed immune responses [2, 38]. Cytokine assessment further supported this result, as both Th1 (IFN-γ, and IL-12) and Th2 (IL-4, and IL-10) cell immune responses were enhanced in mice. The more pronounced changes in IL-12 and IFN-γ levels, which represent Th1 immune responses, reveal that ESPs can induce both humoral and cellular immune responses in mice, characterized by a Th1 bias (increased IgG2a/IgG1 ratio) [5]. Our findings revealed that the Th1-biased response induced by the ES antigens of the plerocercoid differed from the reported Th2-dominant immunity induced by the ES antigens of the plerocercoid [28], but was similar to the mechanism of the Th1-type response and anti-infection protection in *Clonorchis sinensis* infection [36]. This type of a Th1 bias may stem from the abundance of proinflammatory components (*e.g.*, cysteine proteases or mitochondria-associated proteins) in the antigens, which may promote IL-12 secretion by activating the dendritic cell TLR signalling pathway, thereby driving IFN-γ-dependent immunity [6]. Notably, despite marked increases in IL-4 and IL-10 levels (indicating a Th2 response), the simultaneous increase in IL-12 levels and the mild increase in IFN-γ levels support a Th1/Th2-balanced immune pattern. This mixed response may contribute to coordinating the humoral and cellular immune synergy against parasitic infections, while avoiding excessive inflammatory damage. With respect to the results described above, further studies are needed to comprehensively explain and validate the protective effect of ES antigens and identify the functions of key antigens, thereby evaluating their potential as vaccines or diagnostic biomarkers.

## Conclusions

This study revealed the differential expression profiles of adult and plerocercoid ESPs of *S. mansoni* using DIA-based quantitative proteomics technology. Our study identified 580 plerocercoid-specific proteins and revealed 607 DEPs whose expression significantly differed, with functional enrichment in posttranslational modifications, carbohydrate metabolism, and ubiquitination regulatory pathways. For the first time, this study has revealed enrichment of upregulated proteins in neurodegenerative disease-related pathways in plerocercoids, suggesting that simulating host pathologic pathways may enable immune escape. Moreover, the upregulated proteins in adults are involved mainly in the formation of adhesive plaques and antigen processing, which may be related to their intestinal colonization strategies. In addition, RL40 was identified as a key protein by PPI analysis, and the identification of some ES-specific proteins, *e.g.*, ATG7, and unique immunogenic features (Th1-biased Th1/Th2 mixed immune response) was also observed, which provides valuable insights for the development of new diagnostic biomarkers.

**Abbreviations**
ESP: Excretory-secretory protein;
DEPs: Differentially expressed proteins;
FDR: False discovery rate;
GO: Gene Ontology;
KEGG: Kyoto encyclopedia of genes and genomes;
BP: Biological process;
CC: Cellular component;
MF: Molecular function;
PPI: Protein-protein interaction;
qRT-PCR: Quantitative real-time PCR;
ELISA: Enzyme-linked Immunosorbent Assay;
GAPDH: Glyceraldehyde-3-phosphate dehydrogenase.

## Data Availability

The data supporting the conclusions of this article are included within the article.

## Supplementary materials

*Table S1*. Primers sequences designed for qRT-PCR.

The supplementary material of this article is available at https://www.parasite-journal.org/10.1051/parasite/2026025/olm.
