# Supplementary material for: First analysis of Spirometra mansoni excretory–secretory proteins by the 4D-DIA method
Source: Parasite. 2026 Apr 22;33:26. doi: 10.1051/parasite/2026025 (PMC13104597; doi:10.1051/parasite/2026025)
Supplement: Supplementary file 1 — Table S1. Primers sequences designed for qRT-PCR. [file parasite-33-26-s1.pdf]

Table S1. Primers sequences designed for qRT-PCR.

Stage	Protein name	Gene name	Primer Sequence(5'to3')	Product size（bp）
Plerocercoid	Kazal-type serine protease inhibitor domain protein	*SERJ2_LOCUS21220*	F-GCAGAGCGAGCGAAGAAT<br>R-AAGGTGCCCACAAAGAGC	153
	Proliferation-associated protein 2G4	*PA2G4*	F-GTCTATCGTCCTCAATCCG<br>R-GGTCTTTCCTTCGCCAGT	115
	CATL	*CATL*	F-GCCTTATTGTTCCTGGCTTAT<br>R-TTCGCCTTGCTTCCTGTC	323
	Sarcoplasmic calcium-binding protein	*SCP*	F-AAGGCAAGATTACGAAGG<br>R-TTTGTAGTTTATTAGTGCTCCC	110
	Ras-related protein Rab-5C	*TR151277*	F-GAAGTCAGTCCCGAAATGG<br>R-GCTCCTATGGTAGCCTCCT	140
Adult	Heat shock protein 70	*HSP70*	F-CCAGCAGGCAGATAAGGA<br>R-GGCTTGGTACATCTTCGTTA	154
	Kelch-like protein 20	*KLH20*	F-CCTTACAGCCAGACGACG<br>R-CAGCCGATTAGAACAGATGAG	245
	Twitchin	*Titin*	F-CTTTAATTTGGCTCGCTTGG<br>R-GTAGTCGCCGTTCCGTGT	125
	Actin	*SERJ2_LOCUS11136*	F-GAGCCCAGAATGGAACCG<br>R-GACCCTCCTCCTCAAACG	474
Adult	Arrestin_C domain-containing protein	*SERJ2_LOCUS18981*	F-ACGCAAGAGCCGTACATT<br>R-GGTTTGGATTCACCGAGTT	240
Reference gene	glyceraldehyde-3-phosphate dehydrogenase	*GADPH*	F-AGCAACCTCGTTGATGTCGT<br>R-TGAATTGACCGTGGGTGGAG	97
